# An atypical presentation of intrahepatic perforated cholecystitis: a modern indication to open cholecystectomy. Report of a case

**DOI:** 10.1186/1471-2482-14-6

**Published:** 2014-01-27

**Authors:** Marcello Donati, Antonio Biondi, Francesco Basile, Salvatore Gruttadauria

**Affiliations:** 1 Department of Surgery. General and Oncologic Surgery Unit, Vittorio-Emanuele University Hospital of Catania, via Plebiscito 628, 95122 Catania, Italy

**Keywords:** Intrahepatic perforation, Hepatic abscess, Chronic perforation, Chronic cholecystitis

## Abstract

**Background:**

Intrahepatic gallbladder perforation with chronic liver abscess formation was anecdotically reported in the literature. The aim of this work is to report a case of intrahepatic gallbladder perforation and its atypical clinical presentation.

**Case presentation:**

A 62-year-old male patient came to our observation; his medical history showed intermittent fever up to 39-40°C of about 2 weeks and anorexia, with an overall weight loss of about 12 Kg. Physical examination of the abdomen was negative. An ultrasound of the liver and an abdominal CT angiogram detected a disomogeneous hypoechoic-hypodense area in the 5th segment of the liver. Differential diagnosis between hepatic abscess or gallbladder cancer remained open. A surgical exploration was planned. After laparoscopic exploration, a conversion to open procedure with an atypical resection of the 5th hepatic segment was performed. Histologic examination of the specimen showed an intrahepatic chronic perforation of the gallbladder with intrahepatic abscess.

**Conclusion:**

To the best of our knowledge, 18 cases have been reported in the literature as a Niemeier type I perforation. Clinical presentation, even in its extreme rarity, is more often acute. Differential diagnosis between gallbladder cancer versus liver abscess remains controversial. Open approach is mandatory in such cases.

## Background

Perforation of the gallbladder has become a relatively uncommon complication of the natural history of cholelithiasis during acute cholecystitis (0.8-3.2% in recent reviews) [1]. It usually bursts with an acute abdomen and must be operated as a surgical emergency before causing septic peritonitis. Gallbladder perforations were classified following Niemeir’s proposal of 1934 [2]. Gallbladder can perforate freely in the abdominal cavity or in a neighboring organ causing many different clinical situations [3]. The intrahepatic perforation causing a liver abscess is an extremely rare condition, anedoctically reported in the world literature, even in the rare type II or III perforation (subacute or acute perforation) [4]. Liver abscess caused by gallbladder perforation can be a a life-threatening complication (5.6% mortality) [5]. Current management of intrahepatic or hepatic abscess is to submit the patient to a percutaneous drainage supported by intravenous antibiotic therapy, when abscess size does not exceed 5 cm, otherwise surgical exploration and drainage remains the first line treatment option for pyogenic liver abscess [6,7]. In cases of gallbladder perforation cholecystectomy is, of course, the treatment of choice, even if in these cases the laparoscopic approach can be problematic [8]. The aim of this work is to report on an intrahepatic type I perforation of the gallbladder leading to a chronic hepatic abscess, causing a very rare and atypical clinical picture.

## Case presentation

A 62-year-old male patient came to our observation with an initial diagnosis of clinically silent long-term inguinal hernia. His medical history showed intermittent fever and anorexia had been present for one year. In detail, the patient reported that over the previous year he had had alternating intermittent fever up to 39-40°C of about 2 weeks with quiescence periods of about 20–30 days, every few months together with an overall weight loss of about 12 Kg. These symptoms were not associated with any other abdominal or thoracic signs. Physical examination of the abdomen was negative. The fever disappeared after an empiric antibiotic therapy with third generation cephalosporins (1 gr. for 12 days) was started. Laboratory blood samples obtained when the patient was febrile, showed 12,000 WBC count; liver function tests were in the normal range and serologic screening for Salmonella and Brucella antibodies were negative. Blood cultures, antibiograms and tumor markers were also negative (CEA, CA 19.9).

**Figure 1:**
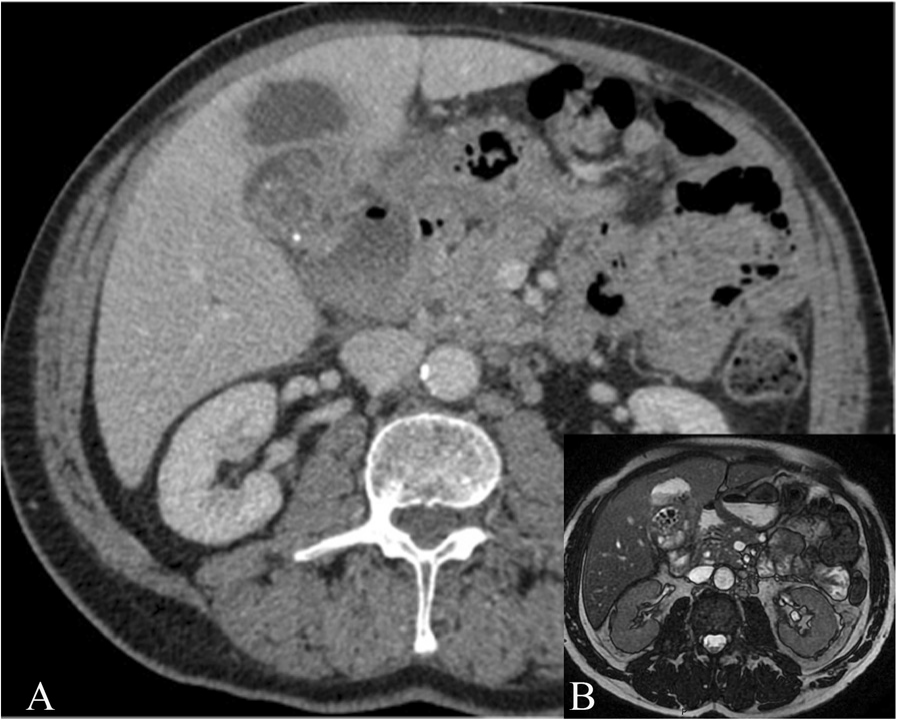
**Preoperative Diagnostic. (A)** Preop.-CT scan. A hypodense pseudocystic mass on the 5th liver segment, mimicking abscessed gallbladder cancer (bile stones are recognizable on the hilar part of the tumor). **(B)** MRI scan. Bubbles of free air inside the “tumour”.

**Figure 2:**
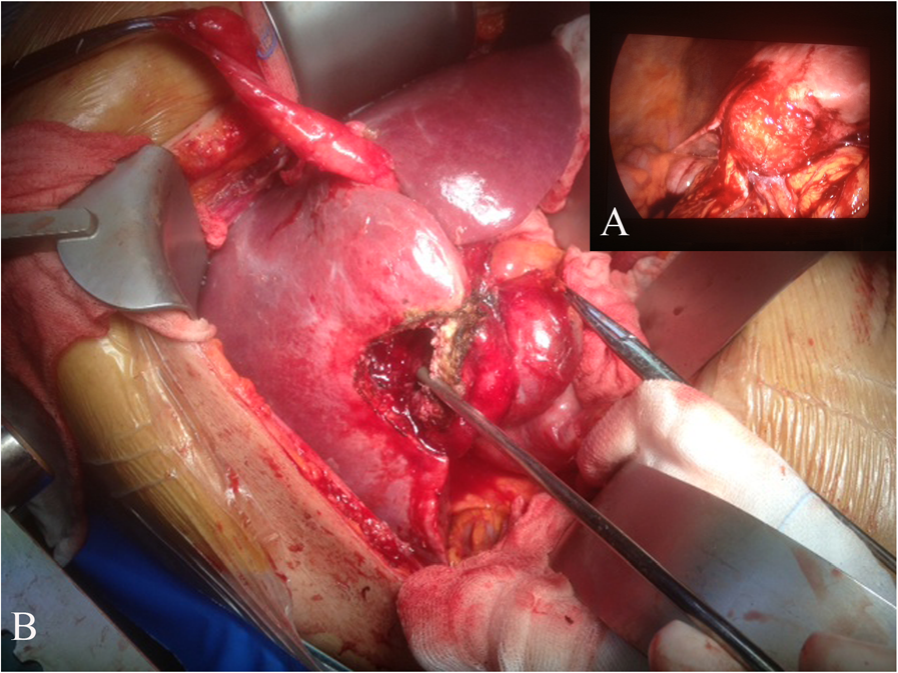
**Intraoperative finding: Intrahepatic perforation of the gallbladder.** Due to strong hilar adhesions and structure visualization difficulties in laparoscopic view **(A)**, a conversion to “open” approach was decided **(B)**.

**Figure 3:**
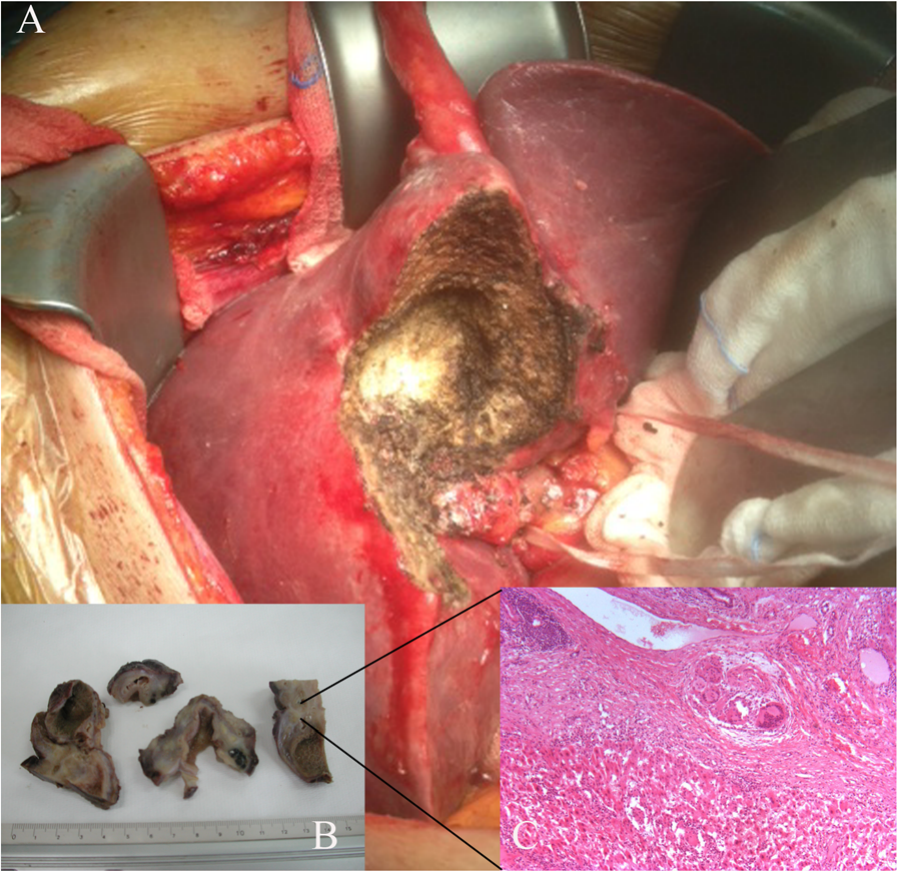
**Specimen examination. A)** A typical partial resection of the 5th Segment *en-bloc* with the gallbladder. Intrahepatic position of the gallbladder. **B)** Macroscopic details of the pathologic specimen: intrahepatic position of the gallbladder. **C)** Microscopic detail of the specimen (Zoom 10 X): chronic inflammatory cells (giant plurinucleated cells) mixed with hepatic regeneration nodes in the context of a granuloma under the gallbladder bed, effect of intrahepatic perforation. (Courtesy of Dr. Loredana Villari. Pathology Institute. Vittorio-Emanuele Hospital. Catania.).

An ultrasound scan of the liver and an abdominal CT scan (Figure 1A) detected a disomogeneous hypoechoic-hypodense mass with many internal hyperechoic/hyperdense stones in the 5th segment of the liver. In addition, a cholelithiasis with two small stones in the common bile duct was present. Differential diagnosis between hepatic abscess or gallbladder cancer remained open. An abdominal MRI scan confirmed the above mentioned findings with the evidence of a few bubbles of free air suggesting spontaneous perforation of the gallbladder (B). An ERCP was then performed and the choledocolithiasis was resolved through a papillotomy. Afterwards a surgical exploration was planned. The operation started with a laparoscopic approach to confirm the suspected diagnosis and exclude tumor diagnosis and even more important peritoneal tumor spread that would be a contraindication to radical R0-surgical resection. Laparoscopy showed a bulging lesion involving the gallbladder, liver, great omentum and duodenum, (Figure 2A) while no signs of peritoneal carcinomatosis were detected. We then decided to convert to open procedure due to uncertain anatomical limits of the lesion in front of the duodenum and the liver hilar structures. After resection of the omental adhesions and the spearing of the duodenum, the bulging was resected en-bloc with the gallbladder (Figures 2B-3) and gallbladder bed performing an atypical resection of the 5th hepatic segment. Opening the large abscess we found more than 20 biliary stones that had migrated intra-hepatically. The phlogistic nature of the tumor was histologically confirmed by three intra-operative sections (later frozen) and on the definitive histologic examination (Figure 3B, C). The post–operative course was uneventful and the patient was discharged on the 4th post-operative day completely afebrile and in good general condition.

Intra-hepatic gallbladder perforation is generally considered to be a very rare evolution of cholelithiasis, in fact, to date, performing a Pubmed search using as key words: “Intrahepatic abscess, intrahepatic gallbladder perforations, Neimeir’s type I perforation, chronic gallbladder perforation” and then using “related articles” and “see reviews” functions of the database, 20 articles were selected but after reading them and avoiding the well-known confusion [9] and mistakes of reporting Neimeir’s classification [4,10], only 18 cases of chronic gallbladder perforation with formation of intrahepatic abscess were found to be reported in the literature [4,11]; therefore following the original classification they should be considered as Niemeier type I perforations (chronic perforation with fistula) [2]. Despite chronic processes with fistula formation, its clinical presentation, even in its rarity, is more often acute (nausea, vomiting, upper quadrant pain, fever, altered mental status, and septic shock) [12,13], or the consequence of an acute perforation [10]. The diagnosis is usually made by US or CT scan. In our case CT scan was not able to exclude a tumor in the 5th hepatic segment. The lack of acute abdomen symptoms characterized our case and this clinical presentation is to be considered exceptional, while usually such a perforation leads to an emergency operation [4]. This subtle clinical picture can be misleading for the physician causing a delay in obtaining prompt imaging tests. Some reports have highlighted the difficult differential diagnosis between gallbladder cancer versus liver abscess [12,14,15]. Because of the very few reports to date, there is no consensus on the standard treatment of such a rare condition [15,16]. Simple puncture and drainage in these cases seems to not be an effective option [15]. An endoscopic examination could be an option, even if in this case laparoscopic cholecystectomy could not be performed due to technical reasons (many adhesions, not clear anatomy, high risk of damage to hilar structures). Some Authors question whether this rare condition is more common in patients with an intrahepatic gallbladder [17]. Therefore chronic liver abscess due to gallbladder perforation is a rare evolution of cholelithiasis.

## Conclusions

Intrahepatic gallbladder perforation with abscess formation should be considered an exceptional clinical entity. Despite new diagnostic tools, differential diagnosis with cancer sometimes remains challenging.

It is our opinion that an open approach or early conversion should, in these cases, as in other Niemeier’s perforation type I [3], still be the preferred surgical treatment.

## Consent

Written informed consent was obtained from the patient for publication of this Case report and any accompanying images. A copy of the written consent is available for review by the Editor of BMC Surgery.

## Abbreviations

Kg: Kilograms; Gr: Grams; CEA: Carcioembriogenic Antigen; WBC: White blood cells; CT: Computed tomography; US: Ultrasound scan; MRI: Magnetic resonance imaging; ERCP: Endoscopy retrograde colangiopancreatography.

## Competing interests

Marcello Donati, Antonio Biondi, Francesco Basile and Salvatore Gruttadauria have no conflict of interests.

## Authors’ contributions

DM wrote the paper and made the final revision. BA collected data and reviewed the text. FB designed the study, chose figures and reviewed the paper. GS coordinated the study, performed the operation, made style and language revision. All authors read and approved the final manuscript.

## Pre-publication history

The pre-publication history for this paper can be accessed here:

http://www.biomedcentral.com/1471-2482/14/6/prepub
